# Azilsartan Suppresses Osteoclastogenesis and Ameliorates Ovariectomy-Induced Osteoporosis by Inhibiting Reactive Oxygen Species Production and Activating Nrf2 Signaling

**DOI:** 10.3389/fphar.2021.774709

**Published:** 2021-11-26

**Authors:** Bin Pan, Lin Zheng, Jiawei Fang, Ye Lin, Hehuan Lai, Jiawei Gao, Wenzheng Pan, Yejin Zhang, Kainan Ni, Chao Lou, Dengwei He

**Affiliations:** ^1^ Department of Orthopedics, Lishui hospital, Zhejiang University School of Medicine, Lishui, China; ^2^ Department of Orthopedic Surgery, Sir Run Run Shaw Hospital, Zhejiang University School of Medicine, Hangzhou, China; ^3^ Key Laboratory of Imaging Diagnosis and Minimally Invasive Intervention Research of Zhejiang Province, Lishui hospital, Lishui, China

**Keywords:** osteoporosis, reactive oxygen species, Nrf2, osteoclast, azilsartan

## Abstract

Osteoporosis is characterized by a decrease in bone mass and destruction of the bone microarchitecture, and it commonly occurs in postmenopausal women and the elderly. Overactivation of osteoclasts caused by the inflammatory response or oxidative stress leads to osteoporosis. An increasing number of studies have suggested that intracellular reactive oxygen species (ROS) are strongly associated with osteoclastogenesis. As a novel angiotensin (Ang) II receptor blocker (ARB), azilsartan was reported to be associated with the inhibition of intracellular oxidative stress processes. However, the relationship between azilsartan and osteoclastogenesis is still unknown. In this study, we explored the effect of azilsartan on ovariectomy-induced osteoporosis in mice. Azilsartan significantly inhibited the receptor activator of nuclear factor-κB ligand (RANKL)-mediated osteoclastogenesis and downregulated the expression of osteoclast-associated markers (Nfatc1, c-Fos, and Ctsk) *in vitro*. Furthermore, azilsartan reduced RANKL-induced ROS production by increasing the expression of nuclear factor erythroid 2-related factor 2 (Nrf2). Mechanistically, azilsartan inhibited the activation of MAPK/NF-κB signaling pathways, while Nrf2 silencing reversed the inhibitory effect of azilsartan on MAPK/NF-κB signaling pathways. Consistent with the *in vitro* data, azilsartan administration ameliorated ovariectomy (OVX)-induced osteoporosis, and decreased ROS levels *in vivo*. In conclusion, azilsartan inhibited oxidative stress and may be a novel treatment strategy for osteoporosis caused by osteoclast overactivation.

## Introduction

Bone homeostasis is mainly sustained by the synergistic actions of two types of cells, namely, osteoblasts for osteogenesis and osteoclasts for osteolysis (Rodan and Martin, 2000; Raisz, 2005). Osteoclasts have been reported to function in promoting bone resorption and maintaining bone homeostasis (Clézardin et al., 2021). However, various pathological conditions may contribute to osteoclast overactivation, including estrogen deficiency in postmenopausal women, inflammation, and oxidative stress (Rodan and Martin, 2000; Manolagas, 2010; Manolagas et al., 2013).

Osteoclasts are identified as multinucleated cells that originate from the monocyte/macrophage lineage and are modulated by receptor activator of nuclear factor-κB ligand (RANKL) and macrophage colony-stimulating factor (M-CSF) (Bruzzaniti and Baron, 2006; Honma et al., 2021). By recruiting TRAF6, RANK further mobilizes the mitogen-activated protein kinases (MAPKs) and nuclear factor (NF)-κB signaling pathways (Walsh et al., 2015), which are critical signals that trigger the transcriptional activity of nuclear factor of activated T cells 1 (NFATc1) and c-Fos in osteoclastogenesis (Boyle et al., 2003; Yamashita et al., 2007; Boutros et al., 2008; Novack, 2011; Tan et al., 2017).

Reactive oxygen species (ROS) are also engaged in bone metabolism (Lean et al., 2003; Sasaki et al., 2009). Upon RANKL stimulation, osteoclast precursors produce endogenous ROS via RANK, TRAF6, Rac1, and Nox1 cascades (Lee et al., 2005). Several studies have confirmed that the inhibition of ROS production prevents osteoclastogenesis (Lee et al., 2005; Chen et al., 2019). Nrf2 is a redox-sensitive leucine zipper transcription factor that decreases ROS levels by promoting the transcription of antioxidant enzymes (Ishii et al., 2000), including glutathione S-transferases (GSTs), heme oxygenase-1 (HO-1), superoxide dismutase (SOD) 1, NAD(P)H: quinone oxidoreductase (NQO) 1, and Catalase (Nioi et al., 2003; Sun et al., 2015). Nrf2 deficiency resulted in increased intracellular ROS levels, defective antioxidant enzyme transcription, and significantly increased osteoclast formation (Hyeon et al., 2013).

Angiotensin (Ang) II, an octapeptide component of the renin-angiotensin system (RAS), exerts its effects through two pharmacologically different G protein-coupled receptors: AT1R and AT2R (de Cavanagh et al., 2004; Benigni et al., 2010). Previous studies have documented the local expression of AT1R in the skeletal system of humans and animals (Zhang et al., 2019). Several components of the RAS, such as AT1R, AT2R, and ACE, are upregulated in mouse calvarial tissue during titanium-induced osteolysis (Zhao et al., 2021). In addition, Yutaro et al. reported that AT1R was existed in osteoblasts, BMMs, preosteoclasts and mature osteoclasts (Asaba et al., 2009). According to recent studies, angiotensin II (Ang II) is involved in bone metabolism and oxidative stress by binding to the AT1 receptor. Deletion of AT1aR increases the number and volume of bone trabeculae and attenuates oxidative stress (Benigni et al., 2009; Kaneko et al., 2011).

Azilsartan, a blocker of the AT1R receptor (ARB), has been approved for clinical use, and it inhibits AT1R-mediated biological effects. Azilsartan restores endothelial function in the inflammatory response by suppressing inflammation and increasing e-NOS phosphorylation (Matsumoto et al., 2014; Liu et al., 2016; Lei et al., 2021). Meanwhile, azilsartan inhibits LPS-induced inflammatory responses in macrophages by suppressing oxidative stress (Dong et al., 2021). However, the effect of azilsartan on osteoclastogenesis has not been evaluated.

In our study, we found that azilsartan inhibited osteoclast formation and resorption function *in vitro* and ameliorated osteoporosis in ovariectomy (OVX) mice. We firstly find that azilsartan may be a promising drug candidate in the treatment for skeletal disorders associated with osteoclasts.

## Materials and Methods

### Animal Ethics

Six-to eight-week-old C57BL/6J mice were purchased from Shanghai Silaike Experimental Animals Center. All mice were housed in cages with ventilation filters under natural light and were provided food and water without any restrictions. The temperature in the room was maintained at 23°C. All animal experiments were approved by the Ethics Committee of Lishui Hospital (Zhejiang University, Zhejiang, China) and were conducted according to the Guide for the Care and Use of Laboratory Animals (National Institutes of Health, China).

### Reagents and Antibodies

Azilsartan was obtained from Selleck Chemicals (Selleck Chemicals, Houston, TX, United States). Azilsartan was dissolved in DMSO (Sigma–Aldrich, Sydney, Australia) and stored at −20°C until use. Fetal bovine serum (FBS) and α-MEM were purchased from Gibco (Gaithersburg, MD, United States). Recombinant M-CSF and RANKL were purchased from R&D Systems (Minneapolis, MN, United States). Specific antibodies against c-Fos, CTSK, NFATc1, GAPDH, β-actin and HO-1 were purchased from Abcam (Cambridge, United Kingdom). Antibodies against p-JNK, JNK, p-ERK, ERK, p-P38, P38, p-IκBα, IκBα, p-P65, P65, and Nrf2 were purchased from Cell Signaling Technology (Danvers, MA, United States). Cell Counting Kit-8 (CCK-8) was purchased from Solarbio Science and Technology (Solarbio, Beijing, China).

### Mouse Bone Marrow-Derived Monocytes/Macrophages Isolation and Culture *in vitro*

We isolated bone marrow-derived monocytes/macrophages (BMMs) from bone marrow flushes of mouse lower limb long bones using a previously published protocol (Zhao et al., 2019). Then, BMMs were cultured in complete α-MEM containing 10% FBS, 25 ng/mL M-CSF, and 100 U/ml penicillin-streptomycin. After 4–5 days in culture, the BMMs were collected for experimental purpose.

### Cytotoxicity Assay

Cell proliferation and cytotoxicity were assessed using CCK-8 assays according to the manufacturer’s protocol. Briefly, 2×10^4^ BMMs were seeded on the surface of 96-well plates, cultured in α-MEM for 24 h, and then treated with various concentrations of azilsartan. At 48 h and 96 h, the medium was replaced with 10 µL of CCK-8 reagent and 90 µL of complete α-MEM for 2 h in a 37°C incubator. Then, the optical density value of each well was measured at 450 nm.

### TRAP Staining and Bone Pit Assay

We tested the effect of azilsartan on osteoclastogenesis and bone resorption function by performing TRAP staining and bone pit formation assays. Briefly, 1 × 10^5^ BMMs were seeded into 24-well plates and cultured with α-MEM containing M-CSF for 48 h. Then, different concentrations (0, 0.25, 0.5, or 1 μM) of azilsartan were added to the complete α-MEM containing RANKL (100 ng/ml) and M-CSF for the indicated periods, and the complete medium was replaced every 2 days. At last, cells were flushed 3 times with phosphate-buffered saline (PBS) and then fixed with 4% paraformaldehyde (PFA) for 20 min at room temperature. Finally, these cells were prepared for TRAP staining. TRAP-positive multinucleated cells with ≥3–5 nuclei were identified as mature osteoclasts.

The bone resorption function of osteoclasts was assessed by bone pit assay. 1 × 10^5^ BMMs were seeded on collagen-coated 6-well plates (Corning, Inc., NY, United States) first and then stimulated with M-CSF (25 ng/ml) and RANKL (100 ng/ml) until small osteoclasts formed. Small osteoclasts were released gently from collagen-coated 6-well plates using the cell dissociation solution (Sigma, MO, United States) and reseeded in equal numbers on Corning hydroxyapatite-coated plates (Corning Inc., NY, United States). Small osteoclasts were cultured in complete α-MEM containing RANKL (100 ng/ml) and different concentrations (0, 0.25, 0.5, or 1 μM) of azilsartan for 2–3 days to observe osteoclast-mediated bone resorption. Finally, the plates were soaked in 5% sodium hypochlorite solution for 2 min followed by washes with purified water until all cells were removed from plate. The bone resorption areas were photographed using Olympus light microscope (Olympus Life Science, Tokyo, Japan). Quantitative analysis was performed by ImageJ software (NIH; Bethesda, MD, United States).

### Total RNA Isolation and qRT–PCR Analysis

Briefly, BMMs were seeded in 12-well plates (2× 10^5^ cells per well) and cultured in α-MEM containing M-CSF (25 ng/ml), RANKL (100 ng/ml) and different concentrations of azilsartan for 5 days. Total cellular RNA was extracted from osteoclasts using an Ultrapure RNA Kit (CWBIO Inc., Beijing, China) in accordance with the protocol. Next, 1 μg of total mRNA was reverse transcribed to cDNAs using a HiFiScript cDNA synthesis kit (CWBIO Inc., Beijing, China). Real-time quantitative PCR was performed using SYBR Green qPCR Master Mix (Yeasen, Shanghai, China) and an ABI 7500 machine (Thermo Fisher Scientific). Relative gene expression was normalized to the expression of β-actin or GAPDH using the 2^−ΔΔCt^ method. All primer sequences are listed in Supplementary Table S1.

### Cell Transfection

Small interfering RNAs (siRNAs) that specifically target mouse Nrf2 gene were designed by RiboBio (RiboBio, Guangzhou, China). The siNrf2 sequences are listed in Supplementary Table S1. SiNrf2 were transfected into BMMs using Lipofectamine 3,000 (Invitrogen, CA, United States) in accordance with the manufacturer’s instructions. Briefly, 5× 10^4^ BMMs were seeded in each well of 24-well plates the day before transfection. After 48 h of incubation, BMMs were transfected with 20 nM siRNA. After 6 h, the medium containing transfection reagent was replaced with complete α-MEM containing M-CSF and RANKL (100 ng/ml). Forty-eight hours later, total mRNA was extracted for quantitative PCR analysis; 72 h later, total proteins were collected for Western blot analysis.

### Western Blot Analysis

Total cellular protein was extracted from osteoclasts using RIPA buffer (CWBIO Inc., Beijing, China) containing PMSF (1%). Ten micrograms of total protein were separated on SDS–PAGE gels and transferred to PVDF membranes (Bio–Rad, Hercules, United States). Then, the membranes were blocked with milk (5%) in TBST for 1 h and incubated with the primary antibody (1:1,000) with shaking overnight at 4°C. After 16 h of incubation, the membranes were rinsed with Tris-Buffered Saline containing Tween-20 (TBST) for 3 times and incubated with a horseradish peroxidase (HRP)-conjugated secondary antibody for 1 h at room temperature. Antibody activities were detected with enhanced ECL hypersensitive chemical luminescence reagents (Yeasen, Shanghai, China). Images were acquired using an Invitrogen iBright 1,500 instrument (Thermo Fisher Scientific) and analyzed using ImageJ software.

### Intracellular Reactive Oxygen Species Detection

Intracellular and intramitochondrial ROS activity were measured using a DCFH-DA probe (Yeasen, Shanghai, China) and a MitoSOX Red assay kit (Yeasen, Shanghai, China). Briefly, BMMs were stimulated with or without RANKL and cultured with different concentrations (0, 0.5, or 1 μM) of azilsartan for 24 h. Then, the probe (5 μM) was added to each well and incubated at 37°C for 20 min. Next, the wells were washed 3 times with cold PBS, and the fluorescence was observed with the fluorescence microscope (Olympus Life Science, Tokyo, Japan). The fluorescence intensity was measured by ImageJ Software.

### Mouse OVX-Induced Osteoporosis Model

A mouse ovariectomy (OVX)-induced osteoporosis model was adopted to evaluate the effect of azilsartan *in vivo*. Ten weeks old mice were randomly divided into 4 groups (*n* = 6 mice per group): sham group (ovaries were only exteriorized but not resected), vehicle group (bilateral ovariectomy + normal saline gavage), low-dose group (bilateral ovariectomy +1 mg/kg azilsartan gavage) and high-dose group (bilateral ovariectomy + 3 mg/kg azilsartan gavage). The dose of azilsartan used was based on the previous study (Abdelsaid et al., 2014; Sukumaran et al., 2017). All mice were anaesthetized by administering an intraperitoneal injection of pentobarbital (40 mg/kg body weight). Mice with a uniform heartbeat and respiration, relaxed muscles, no movement of limbs and, no touching reaction of whiskers were considered to have reached the state of complete anesthesia. The surgical procedure was performed as previously described (Xu et al., 2021). Azilsartan was administered by oral gavage twice a week for 6 weeks beginning on the seventh postoperative day. All mice were euthanized under anesthesia after 6 weeks of azilsartan intervention. The serum sample was collected from each mouse for liver functional enzyme analysis before the mice were euthanized. Then, all femurs and tibias of mice were harvested for the following experiments.

### μCT and Image Reconstruction

After fixation with 4% PFA for 48 h, the mouse right tibia and femur were scanned using a SkyScan 1,275 micro-CT (Bruker, Billerica, MA, United States). Data were analyzed using the following conditions: 50 kV, 9 μm resolution and 75 μA. All images were reconstructed with SkyScan CTAn software. The quantitative analysis was performed within a region set at 150 layers below the growth plate. Related parameters, including trabecular thickness (Tb. Th), bone volume per tissue volume (BV/TV), trabecular number (Tb. N), and trabecular separation (Tb. Sp), were recorded and analyzed.

### Bone Histomorphometric Analysis

All tibias were decalcified in 10% ethylenediaminetetraacetic acid (EDTA) (Sigma, Australia) at 4°C for 14 days. Tibias were dehydrated, embedded in paraffin blocks, and sectioned using a microtome at a thickness of 5 µm. Then, the bone sections were subjected to hematoxylin and eosin (H&E) and TRAP staining. The number of TRAP-positive osteoclast and osteoclast surfaces per bone surface (Oc.S/BS) was assessed in each sample using ImageJ software.

ROS levels were detected *in vivo* using a dihydroethidium (DHE) (Yeasen, Shanghai, China) probe. Briefly, fresh left tibias were fixed with 4% PFA at 4°C for 4 h. EDTA was applied for decalcification for 12 h and then replaced with a cryoprotectant solution. All tissues were stored in the cryoprotectant solution for another 24 h. Finally, bone sections were frozen. Sections with a thickness of 5 μm were prepared. Cell nuclei were stained with DAPI. Six random regions per group were quantified using ImageJ software.

### Statistical Analyses

In general, all data were recorded from three or more independent experiments and presented as the means ± SD. The results were analyzed using one-way analysis of variance (ANOVA) with Tukey’s post hoc test or Student’s t test with GraphPad Prism 7 software. *p* < 0.05 was considered statistically significant.

## Results

### Azilsartan Inhibits RANKL-Induced Osteoclastogenesis *in vitro*

**FIGURE 1:**
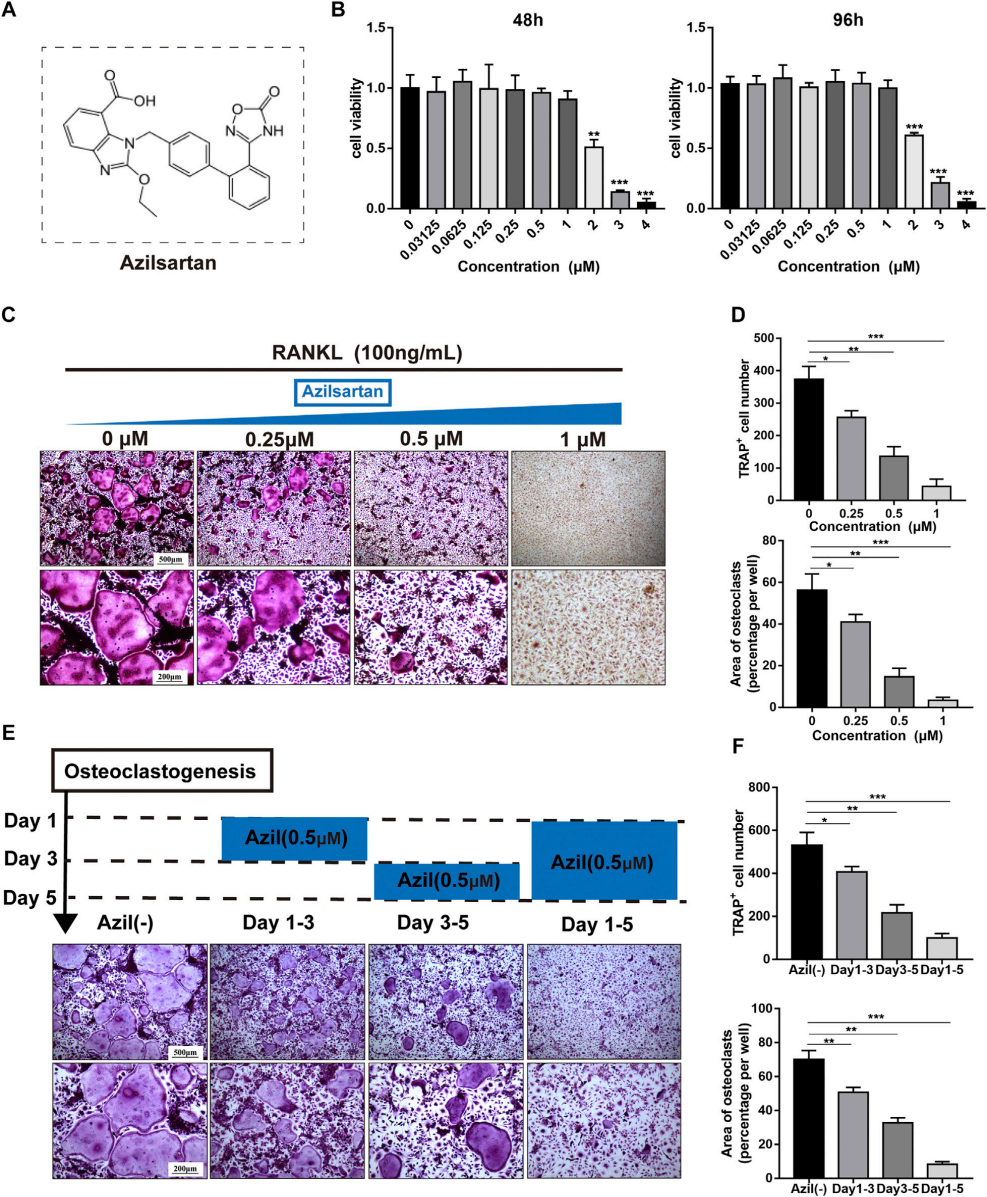
Azilsartan inhibits RANKL-induced osteoclastogenesis *in vitro*. **(A)** The chemical structure of Azilsartan. **(B)** CCK-8 cytoxicity assay was performed to assess the cytotoxic effect of Azilsartan in BMMs at 48 h and 96 h. **(C)** BMMs were treated with M-CSF (25 ng/ml), RANKL (100 ng/ml) and various concentrations of Azilsartan for 5 days. Then the cells were fixed with 4% PFA and stained for TRAP. **(D)** Quantitative analysis of TRAP positive multinucleated cells (nuclei >3) number and the area per well (%) (*n* = 3). **(E)** TRAP staining images showing BMMs were treated with 0.5 μM Azilsartan for indicated days during osteoclastogenesis. **(F)** Quantitative analysis of each group TRAP positive multinucleated cells in different time periods (*n* = 3). All data were obtained from three independent experiments and were shown as mean ± SD; (*p < 0.05, **p < 0.01, ***p < 0.005, ns, no significance, compared with the untreated control group).

The chemical structure of azilsartan is shown in Figure 1A. We performed CCK-8 cell proliferation and cytotoxicity assays in 96-well plates after BMMs were treated with various concentrations of azilsartan to detect the cytotoxicity of azilsartan toward BMMs. As shown in Figure 1B, azilsartan had no effect on cell viability at concentrations ranging from 0 to 1 μM (48 h and 96 h). Next, TRAP staining results showed the formation of a large number of TRAP-positive multinucleated osteoclasts in the control group (0 μM), while osteoclastogenesis was inhibited in a dose-dependent manner by increasing concentrations of azilsartan (Figures 1C,D).

We treated BMMs with 0.5 μM azilsartan at different times to explore which stage of osteoclastogenesis was affected. Interestingly, osteoclast formation was more significantly inhibited when azilsartan was added at the mid-late stage (Days 3–5), while the inhibitory effect was not apparent at the early stage (Days 1–3) (Figures 1E,F).

### Azilsartan Affects Osteoclast Resorption Function and Suppresses Osteoclast-Specific Gene Expression

**FIGURE 2:**
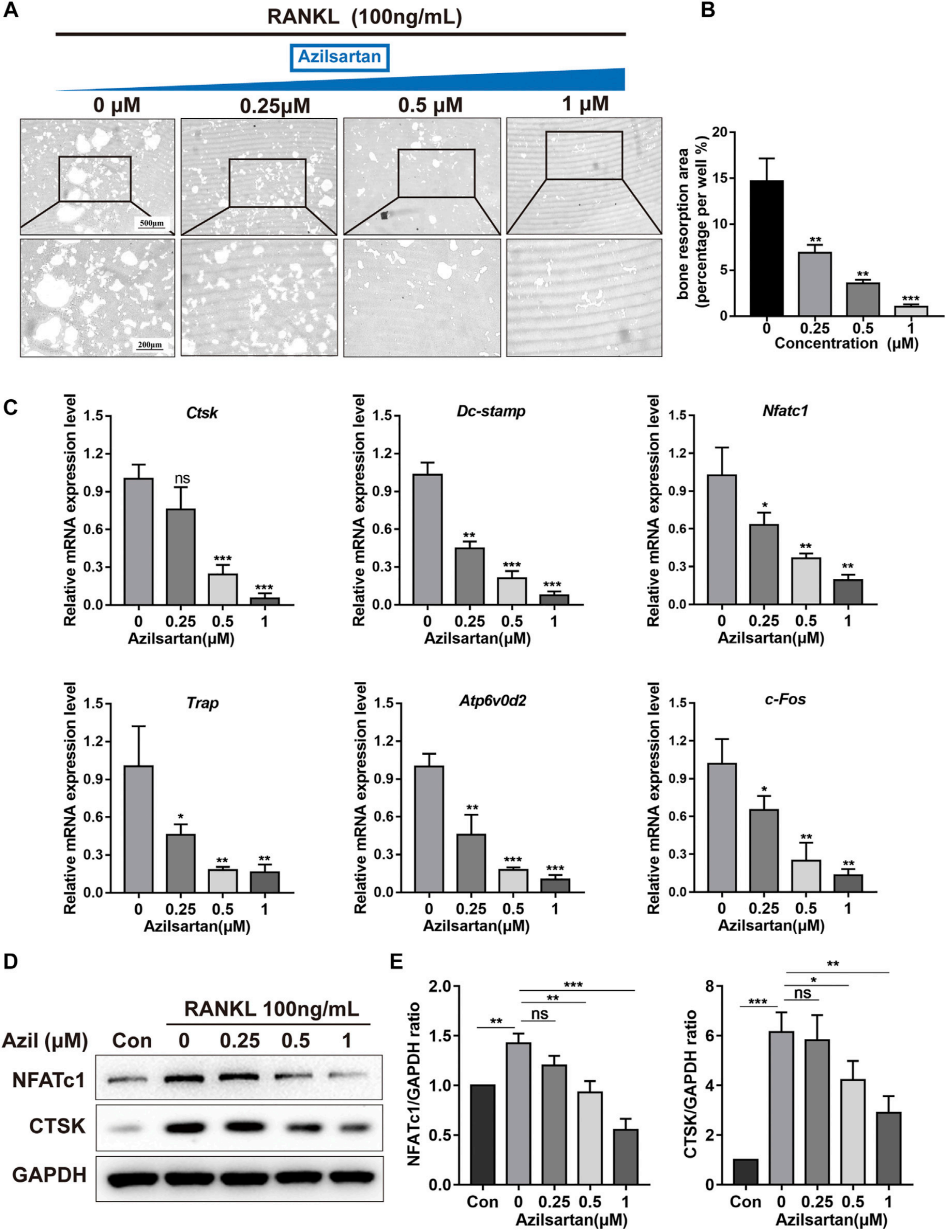
Azilsartan inhibits osteoclast resorption function and affects osteoclast-specific genes expression. **(A)** Representative images of osteoclast bone resorption on the hydroxyapatite surface; BMMs were treated with RANKL (100 ng/ml) for 3 days until small mature osteoclasts formed. Then, osteoclasts were reseeded and cultured with different concentrations of Azilsartan for another 3 days in hydroxyapatite-coated plates. **(B)** Quantitative analysis of the bone resorption area per well using the ImageJ software(*n* = 3). **(C)** The specific-genes expression of *Nfatc1*, *Ctsk*, *Trap*, *c-Fos*, *Atp6v0d2*, and *Dc-stamp* were determined by qPCR; BMMs were stimulated with RANKL and different concentrations of Azilsartan (0, 0.25, 0.5, and 1 μM) for 5 days (*n* = 3). Untreated cells were used as a control. **(D)** Representative Western Blot images showing the protein expression levels of NFATc1, CTSK in osteoclasts. BMMs were stimulated with RANKL (100 ng/ml) and indicated concentrations of Azilsartan (0, 0.25, 0.5, and 1 μM) for 5 days. Then total cellular proteins were extracted and subjected to Western Blot analysis. **(E)** Quantitative analysis of the ratios of protein expression levels of NFATc1 and CTSK relative to GAPDH. All data were obtained from three independent experiments and were shown as mean ± SD, (*p < 0.05, **p < 0.01, and ***p < 0.005, ns, no significance, compared with the 0 μM Azilsartan group).

We next tested whether azilsartan affected osteoclast bone resorption function. Mature small osteoclasts were reseeded on the surface of Corning hydroxyapatite-coated plates and treated with different concentrations of azilsartan (0, 0.25, 0.5, or 1 μM) for 3 days. As shown in Figure 2A, azilsartan treatment significantly reduced the bone resorption area in a dose-dependent manner compared with that of the untreated control group (Figure 2B).

Several osteoclast markers, including Cathepsin K (CTSK), Tartrate Resistant Acid Phosphatase (TRAP), Dendritic Cell-Specific Transmembrane Protein (DC-STAMP), D2 isoform of vacuolar ATPase Vo domain (ATP6v0d2), NFATc1, and c-Fos are upregulated during RANKL-induced osteoclastogenesis (Zheng et al., 2019; Yang et al., 2021). Our results revealed that azilsartan significantly suppressed the expression of these genes compared with the untreated control group (Figure 2C). Next, we investigated the expression levels of proteins associated with osteoclastogenesis and osteolysis, namely, NFATc1 and CTSK, using Western blot analysis. Consistent with the quantitative PCR results, different concentrations of azilsartan exerted an inhibitory effect on osteoclastogenesis and bone resorption, but were not toxicity effect of azilsartan (Figures 2D,E).

### Azilsartan Inhibits the RANKL-Induced Activation of the NF-κB and MAPK Pathways

**FIGURE 3:**
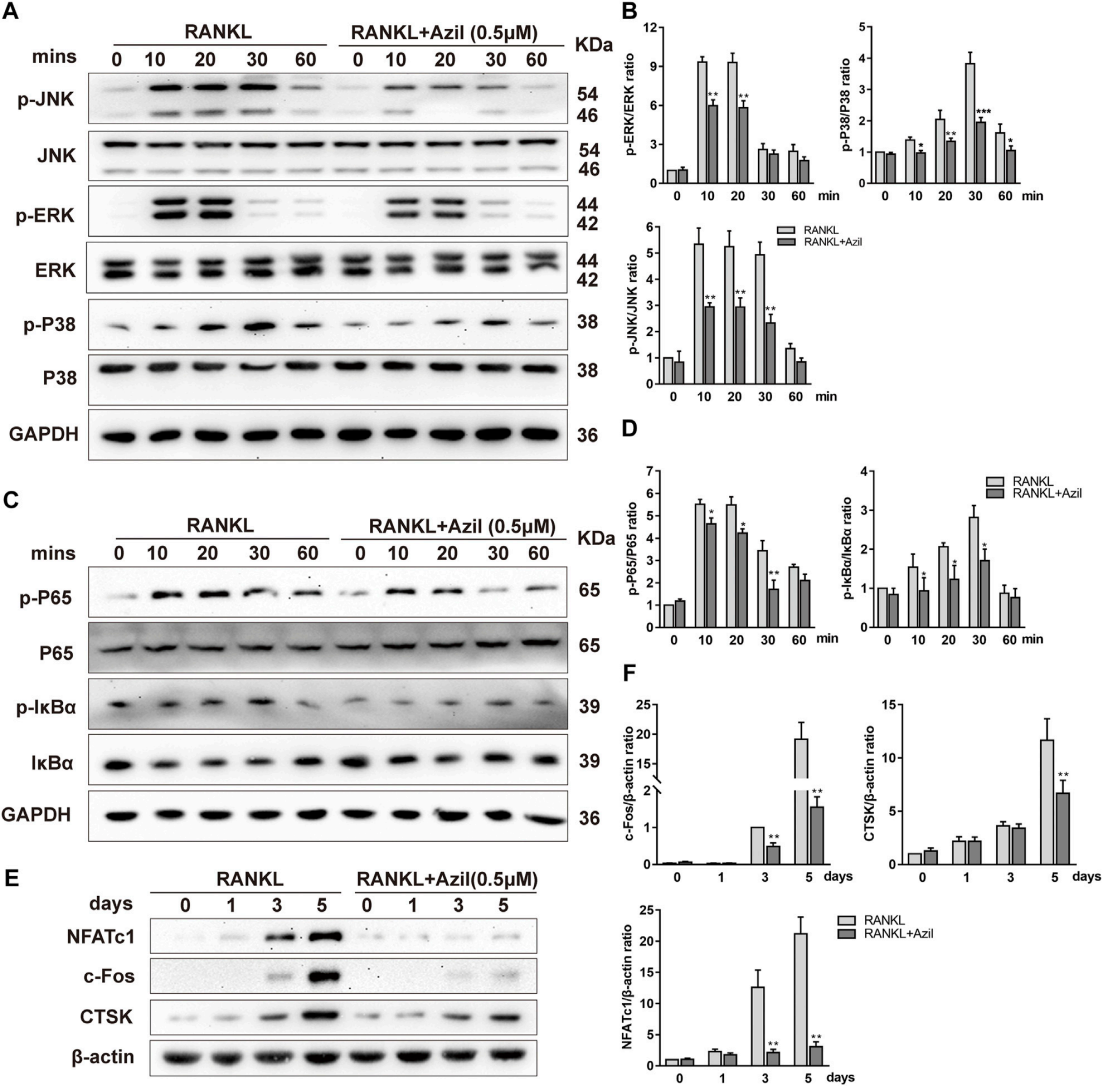
Azilsartan inhibits RANKL-induced activation of NF-κB and MAPK Pathways *in vitro*. **(A)** BMMs were starved and pretreated with Azilsartan (0.5 µM) for 2 h before being stimulated by RANKL. Then, RANKL (100 ng/ml) was added at the indicated time points (0, 10, 20, 30, and 60 min). Total cellular proteins were collected and the phosphorylated and total P38/ERK/JNK were detected by Western Blot. **(B)** Quantitative analysis of phosphorylated P38/ERK/JNK band intensity relative to total P38/ERK/JNK using the ImageJ software (*n* = 3). **(C)** Western blot images showing the effects of Azilsartan on activation of NF-κB signaling, including p-P65/p-IκBα. **(D)** Quantitative analysis of phosphorylated P65/IκBα band intensity relative to total P65/IκBα (*n* = 3). **(E)** Western blot images showing the protein expression levels of NFATc1, c-Fos, and CTSK during osteoclastogenesis. BMMs were treated with RANKL (100 ng/ml) and the indicated concentration of Azilsartan (0.5 µM) for 0, 1, 3, and 5 days; the total protein were extracted for Western Blot analysis. **(F)** Quantitative analysis of the band intensity of NFATc1, c-Fos and CTSK relative to β-actin using the ImageJ software (*n* = 3). All data were obtained from three independent experiments and were shown as mean ± SD. (*p < 0.05, **p < 0.01, ***p < 0.005, compared with the RANKL group).

We identified the molecular mechanisms by which azilsartan inhibits osteoclastogenesis by examining the major signaling pathways affecting osteoclastogenesis, including NF-κB and MAPK. Western blot analyses indicated that both NF-κB and MAPK signaling were activated by RANKL stimulation. However, azilsartan treatment reduced p65 phosphorylation and IκBα phosphorylation, accompanied by the inhibition of IκBα degradation at 10–30 min (Figures 3C,D). Regarding the MAPK signaling pathway, the phosphorylation of P38, ERK, and JNK was significantly reduced after azilsartan treatment (Figures 3A,B), indicating a failure to activate MAPK signaling.

Furthermore, the expression of NFATc1/c-Fos, the essential transcriptional regulator involved in RANKL-induced osteoclastogenesis, was significantly suppressed on days 3 and 5 of osteoclastogenesis in BMMs treated with azilsartan (Figures 3E,F). Cathepsin K, which is considered a downstream protein of exercise-induced resorption function, was inhibited by azilsartan treatment (Figures 3E,F). In summary, our results showed that azilsartan suppressed osteoclastogenesis by inhibiting the NF-κB and MAPK pathways.

### Azilsartan Inhibits RANKL-Induced ROS Production and Increases the Expression of Nrf2

**FIGURE 4:**
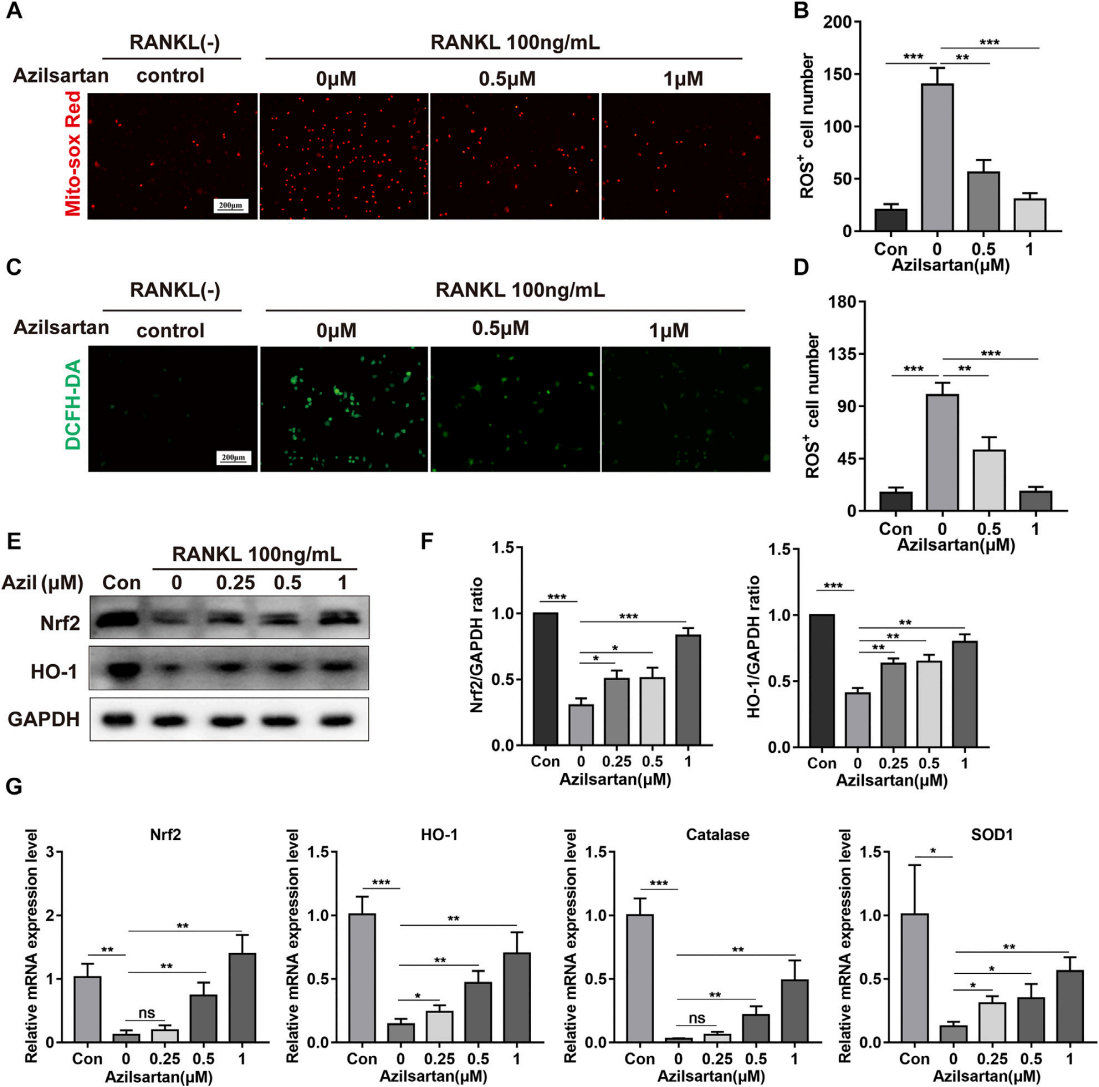
Azilsartan inhibits RANKL-induced ROS production and activates the Nrf2/HO-1 signaling. **(A)** BMMs were treated with different concentrations of Azilsartan (0, 0.5 and 1 μM) for 48 h, and then intramitochondrial ROS was detected by MitoSOX Red probe. **(B)** Quantitative analysis of MitoSOX Red fluorescence (red) intensity (*n* = 3). **(C)** Representative images showing RANKL-induced Intracellular ROS generation in BMMs. **(D)** Quantitative analysis of DCFH-DA fluorescence (green) intensity (*n* = 3). **(E)** Western blot images showing the effect of Azilsartan on Nrf2/HO-1 signaling. **(F)** Quantitative analysis of the band intensity of Nrf2 and HO-1 relative to GAPDH using the ImageJ software (*n* = 3). **(G)** The specific mRNA expression of *Nrf2*, *Ho-1*, *Catalase*, *Sod1* (*n* = 3). All data were obtained from three independent experiments and were shown as mean ± SD. (*p < 0.05, **p < 0.01, ***p < 0.005, ns, no significance, compared with the RANKL + 0 μM Azilsartan group).

ROS play a critical role in osteoclast formation (Lean et al., 2003), and previous studies have revealed that angiotensin II (Ang II) and AT1R are associated with intracellular ROS production (Benigni et al., 2010; Chao et al., 2018). Next, we examined the ROS level in osteoclast mitochondria using the MitoSOX Red probe. RANKL stimulation significantly increased the ROS level in mitochondria. However, azilsartan treatment resulted in a dose-dependent decrease in ROS levels in mitochondria (Figures 4A,B). Similarly, ROS levels in the cytoplasm were significantly reduced by azilsartan, as detected with the DCFH-DA fluorescence probe (Figures 4C,D).

Nrf2, a critical protein in the antioxidant stress system, regulates oxidative stress and ROS production by binding to antioxidant response elements (AREs) and promoting the transcription of downstream antioxidant and detoxification enzymes (Sun et al., 2015). In our study, the expression of Nrf2 and HO-1 was increased in the presence of azilsartan (Figures 4E,F). The expression of other antioxidant enzymes in the cellular antioxidant system, including catalase and SOD1, was increased at the mRNA levels after azilsartan treatment (Figure 4G). In summary, azilsartan exhibited potent antioxidant properties in osteoclasts.

### Azilsartan Suppresses RANKL-Induced NF-κB/MAPK Signaling by Regulating Nrf2 *in vitro*

**FIGURE 5:**
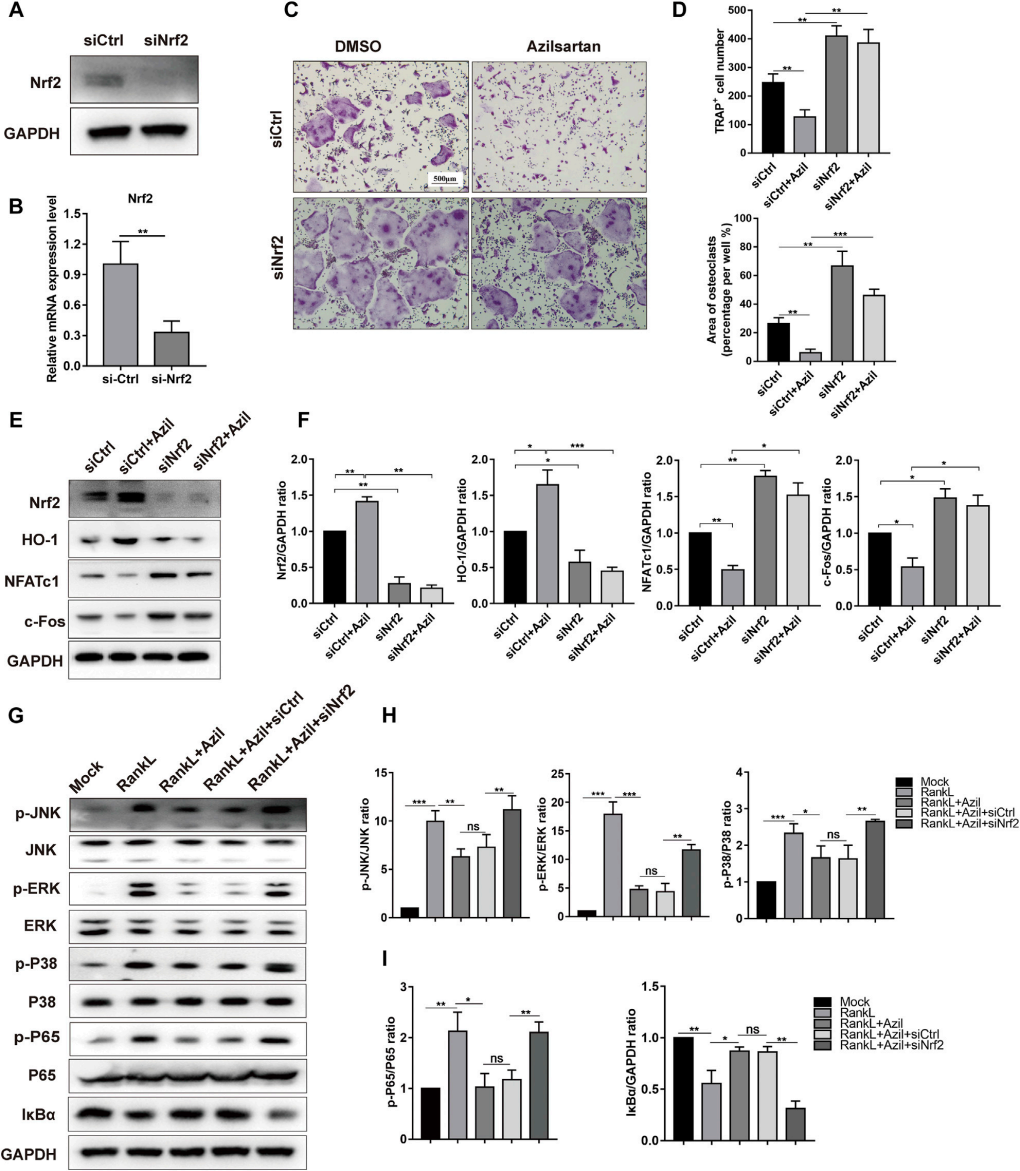
Azilsartan suppresses RANKL-induced osteoclastogenesis *in vitro via* Nrf2. **(A,B)** RNAi of Nrf2 was generated by siRNA in BMMs. **(C)** BMMs were transfected with siNrf2 to reverse the inhibitory effect of Azilsartan in osteoclastogenesis. TRAP staining was performed to detect osteoclast formation. **(D)** Quantitative analysis of TRAP positive multinucleated cells (nuclei >3) number per well and the area per well (%) (*n* = 3). **(E)** Nrf2 silencing could upregulate the expression of NFATc1 and c-Fos, as evidenced by Western Blot analysis. **(F)** Quantitative analysis of the band intensity of Nrf2, HO-1, NFATc1, and c-Fos relative to GAPDH using the ImageJ software. **(G)** The phosphorylated P65, IκBα degeneration and phosphorylated P38/JNK/ERK were detected by Western blot. BMMs were transfected with siNrf2 for 48 h and then pre-treated with Azilsartan for 2 h before being stimulated by RANKL. The total proteins were harvested after 30 min of RANKL (100 ng/ml) stimulation. **(H)** Quantitative analysis of phosphorylated band intensity relative to total ERK/P38/JNK using the ImageJ software (*n* = 3). **(I)** Quantitative analysis of band intensity of p-P65, IκBα relative to P65, GAPDH (*n* = 3). All data were obtained from three independent experiments and were shown as mean ± SD. (*p < 0.05, **p < 0.01, ***p < 0.005, ns, no significance).

Next, we investigated the crosstalk among azilsartan, Nrf2, and osteoclastogenesis. A Nrf2-specific siRNA was transfected into BMMs. The efficiency of transfection was evaluated using Western blot analysis and quantitative PCR analysis, as shown in Figures 5A,B. Compared with the siCtrl group, a larger osteoclast area was observed in the siNrf2 group, and siNrf2 transfection diminished the inhibitory effect of azilsartan on osteoclast formation (Figures 5C,D). In addition, we further harvested the total cellular proteins from the aforementioned groups for Western blot analysis. Nrf2 silencing decreased the expression of antioxidant enzyme HO-1 and increased the expression of crucial osteoclast-associated transcription factors (NFATc1 and c-Fos) in osteoclastogenesis (Figures 5E,F). Surprisingly, azilsartan treatment had little effect on reversing siNrf2-mediated osteoclast formation and protein expression (Figures 5C,E), suggesting that Nrf2 likely acts as downstream of azilsartan.

Furthermore, we detected the levels of critical proteins involved in MAPK signaling to explore the underlying mechanisms by which azilsartan alters NF-κB and MAPK signaling. Nrf2 silencing using siNrf2 reversed the low levels of phosphorylated JNK, phosphorylated P38 and phosphorylated ERK in the azilsartan treatment group (Figures 5G,H). SiNrf2 transfection also reversed the repression of NF-κB signaling, as evidenced by increased levels of phosphorylated P65 and IκBα degradation (Figures 5G,I). Collectively, Nrf2 was identified as a downstream target of azilsartan that regulates MAPK and NF-κB signaling pathways in BMMs.

### Azilsartan Administration Prevents OVX-Induced Bone Loss *in vivo*

**FIGURE 6:**
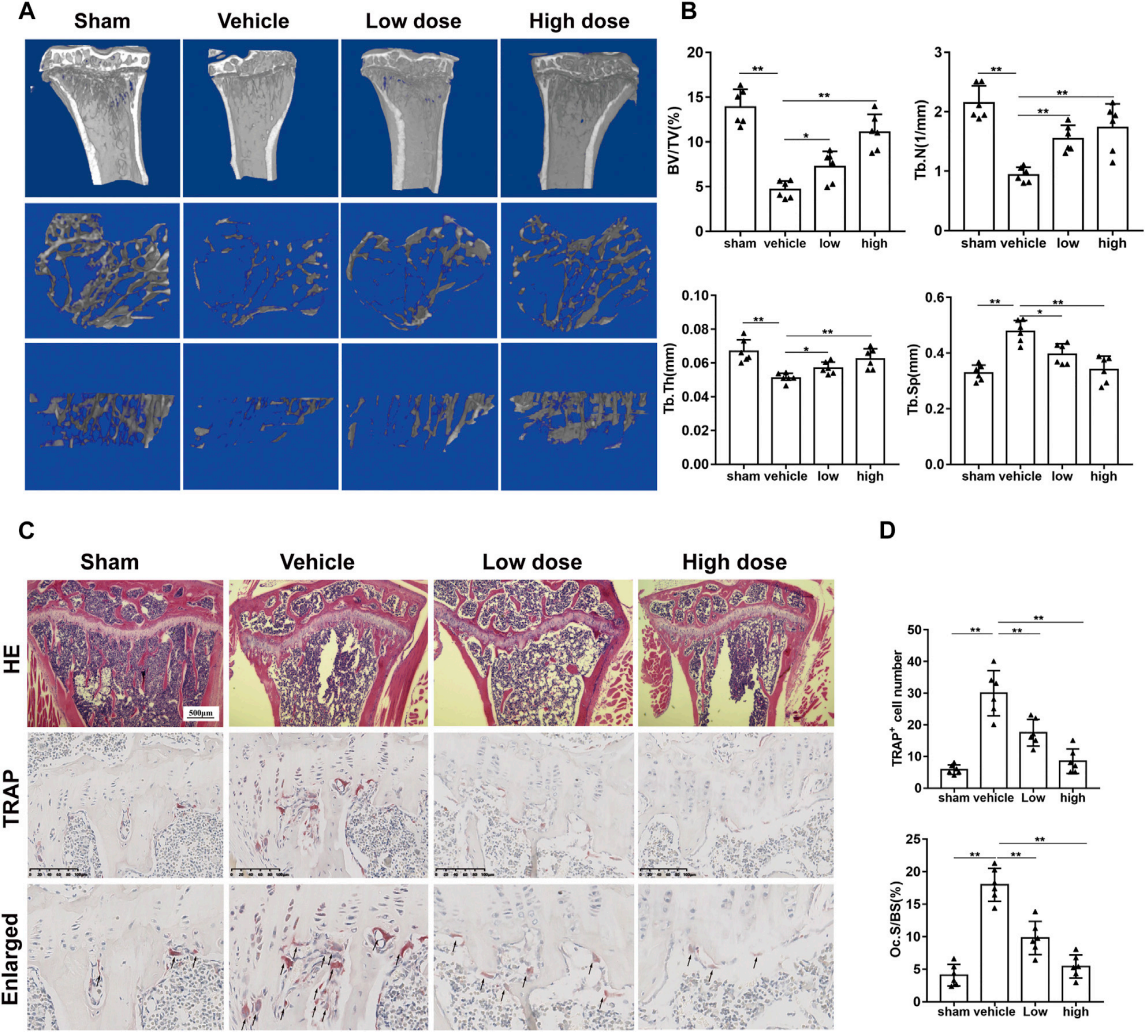
Azilsartan administration prevents OVX-induced bone loss *in vivo*. **(A)** Micro-CT reconstruction of proximal tibial bone of mice from each group: sham-treated (sham), OVX with normal saline gavage (vehicle), OVX with 1 mg/kg Azilsartan gavage (low dose), OVX+ 3 mg/kg Azilsartan gavage (high dose). **(B)** The trabecular thickness (Tb. Th), bone volume/tissue volume (BV/TV), trabecular number (Tb. N), and trabecular separation (Tb. Sp) were measured to evaluate the bone tissue microstructure (*n* = 6 per group). **(C)** Representative H&E (upper panel) and TRAP staining (lower panel) sections from each group. **(D)** Quantitative analysis of the osteoclasts on the surface of trabecular bone and the Oc.S/BS (%) using ImageJ (*n* = 6 per group). All data were shown as mean ± SD. (*p < 0.05, **p < 0.01, ***p < 0.005, compared with the vehicle group).

We established an osteoporosis model in mice to explore the effect of azilsartan *in vivo*. No mouse death or other adverse events occurred during OVX surgery or azilsartan administration. Compared to the sham-operated group, the vehicle group showed significant bone loss. However, the azilsartan-treated group exhibited increased bone mass (Figure 6A). By measuring and comparing the bone parameters, we observed increases in the trabecular number (Tb. N), trabecular thickness (Tb. Th), and bone volume/tissue volume (BV/TV) and a decrease in trabecular separation (Tb. Sp) in the azilsartan-treated group compared with the vehicle group (Figure 6B). Bone histomorphometry results from H&E staining confirmed that azilsartan treatment prevented estrogen deficiency-induced bone loss (Figure 6C). Significant trabecular destruction and bone loss were observed in the OVX groups. Azilsartan administration exerted a protective effect on bone in the low-dose groups and high-dose groups compared with the vehicle groups.

**FIGURE 7:**
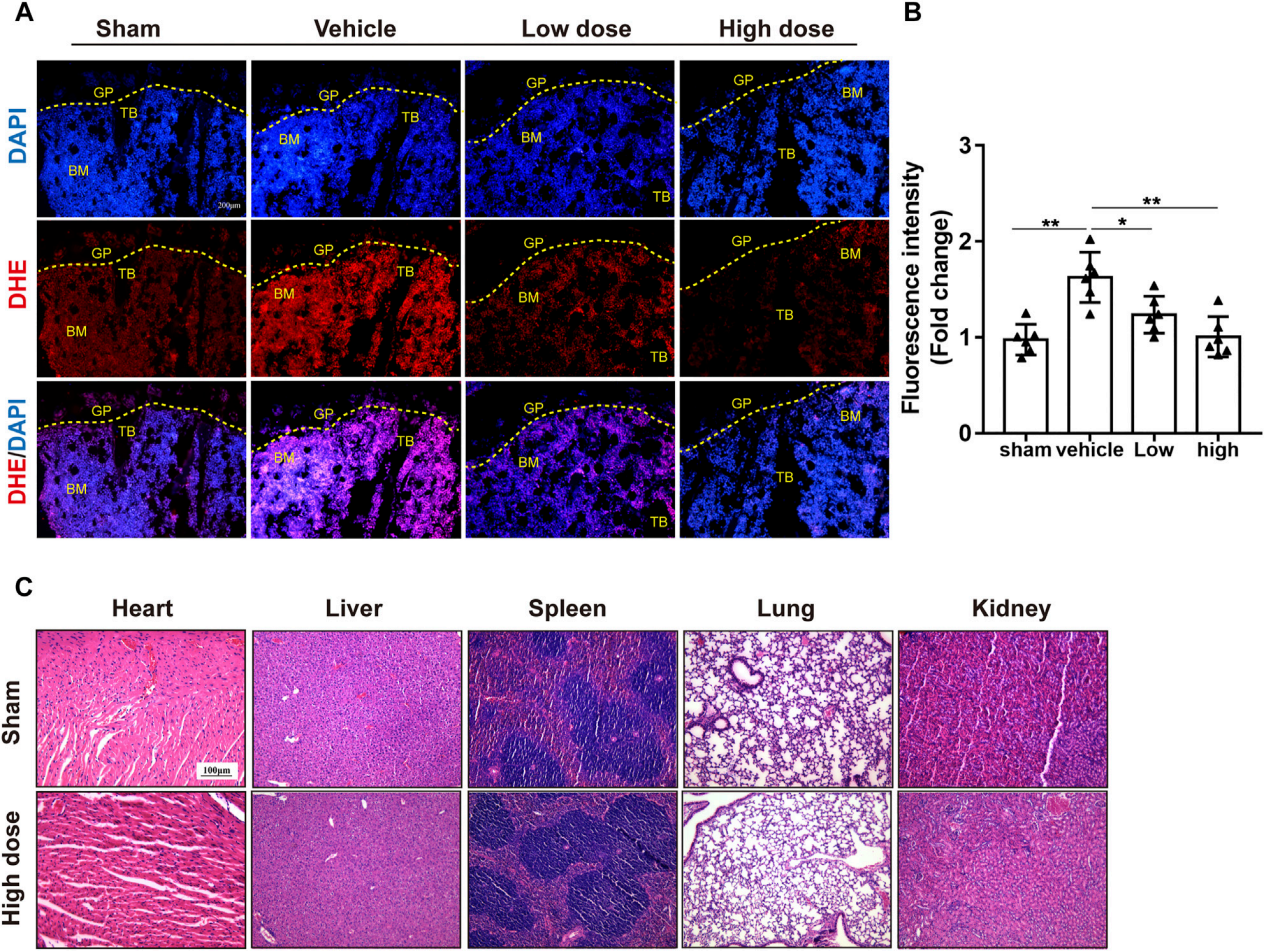
Azilsartan reduced ROS production in OVX mice. **(A)** Representative images of bone cryosections showing ROS production in each group using DHE fluorescence staining. **(B)** Quantitative analysis of ROS fluorescence (Red) intensity (*n* = 6 per group). **(C)** H&E staining of the heart, liver, spleen, lung, and kidney tissues of mice in the Azilsartan-treated and control groups. All data were shown as mean ± SD. (*p < 0.05, **p < 0.01, ***p < 0.005, compared with the vehicle group). TB, trabecular bone; GP, growth plate; BM, bone marrow; DHE, dihydroethidium.

Likewise, TRAP staining showed that azilsartan treatment reduced the number of osteoclasts on each bone surface compared with that of the vehicle groups. The treatment also reduced the TRAP-positive cells/bone surfaces (Oc.S/BS%) ratio (Figures 6C,D). As azilsartan displayed good antioxidant activity *in vitro*, we next detected ROS levels using DHE fluorescent probes in frozen tibial sections. ROS levels within the bone marrow microenvironment were significantly increased after OVX surgery; however, azilsartan treatment reversed this trend (Figures 7A,B).

Furthermore, H&E staining of the lung, heart, spleen, liver, and kidney tissues indicated that azilsartan had no organ toxicity at the administered dose (Figure 7C). Blood biochemistry examinations of mouse serum showed no difference between the sham groups and azilsartan-treated groups (Supplementary Table S2; Supplementary Figure S1). Taken together, azilsartan administration ameliorates OVX-induced bone loss, possibly by inhibiting ROS production.

## Discussion

Osteoporosis, a common bone metabolic disease, has been a major threat to postmenopausal women and the aging population (Compston et al., 2019). Throughout the human lifetime, bone remodeling processes continuously occur. This biological process is mainly regulated by both osteoblasts and osteoclasts. However, excessive activation of osteoclasts in the presence of estrogen deficiency may lead to an imbalance in bone metabolism, resulting in osteoporosis (Boyle et al., 2003). Currently, clinically available drugs for osteoporosis, including hormone replacement, bisphosphonates, and denosumab, are effective but cause some adverse reactions, including gastrointestinal bleeding, atrial fibrillation, and increasing the risk of breast cancer (Shang, 2006; Knopp-Sihota et al., 2013). Therefore, novel alternative drugs that target osteoclasts are also required.

Based on accumulating evidence, ROS play a critical role in osteoclastogenesis (Lean et al., 2003; Agidigbi and Kim, 2019). RANKL induces ROS production in osteoclasts, and accordingly, the inhibition of ROS production inhibits osteoclastogenesis (Chen et al., 2019; Liu et al., 2019). Some drugs that inhibit ROS production in osteoclasts may be novel therapeutic approaches for osteoporosis (Wang et al., 2021). In our study, we first confirmed that azilsartan inhibits osteoclastogenesis by suppressing ROS production *in vitro* and protects against OVX-induced osteoporosis *in vivo*.

Ang II and its receptor (AT1R) have been reported to induce NADPH oxidase (NOX)-dependent oxidative stress, generating ROS in osteoclasts (Nguyen Dinh Cat et al., 2013; Zhang et al., 2019; Zhou et al., 2020). In our present study, we found that azilsartan, an AT1R blocker, suppressed intramitochondrial and intracellular ROS production in osteoclasts. Mice subjected to ovariectomy presented significantly increased ROS levels, while DHE fluorescence probes detected decreased ROS levels when animals were treated with azilsartan *in vivo.* Meanwhile, elevated mRNA levels resulting from stimulation with RANKL, such as transcripts of osteoclastogenesis-related genes (*c-Fos* and *Nfatc1*), genes encoding osteolysis-related enzymes (*Trap* and *Ctsk*), and genes involved osteoclast fusion (*Atp6v0d2* and *Dc-stamp*), were reversed by azilsartan treatment, suggesting impaired osteoclastogenesis and fusion failure. Besides, the results from micro-CT, H&E and TRAP staining confirmed that azilsartan prevented estrogen deficiency-induced bone loss and trabecular destruction *in vivo*.

Previous studies have reported that Nrf2 is involved in regulating osteoclast formation and bone resorption function after its release from Kelch-like ECH-associated protein 1 (Keap1) and translocation into the nucleus, where it binds to AREs and ultimately activates downstream antioxidant enzymes to reduce ROS levels (Kanzaki et al., 2013). Keap1, a cytoplasmic actin-binding protein, encapsulates Nrf2 tethered in the cytoplasm and inhibits Nrf2 transactivation activity, causing the ubiquitination, and degradation of Nrf2 (Sun et al., 2015). Ni et al. found that schisandrin A increases the stability of Nrf2 and inhibits the ubiquitination and degradation of Nrf2 in osteoclasts. Intracellular ROS are the net effect of the balance between ROS generation and Nrf2-mediated clearance of ROS by the intracellular antioxidant system. Some antioxidants, such as pristimerin, chitosan, octyl itaconate, were reported to reduce ROS levels and inhibit osteoclast differentiation by activating Nrf2/HO-1 signaling (Chen et al., 2020; Ni et al., 2020; Qi et al., 2020). Nrf2 activation inhibits osteoclastogenesis, while inactivation of Nrf2 suppresses antioxidant enzyme expression and increases intracellular ROS levels in osteoclasts, subsequently promoting osteoclastogenesis (Kanzaki et al., 2013; Sun et al., 2020; Sánchez-de-Diego et al., 2021). As shown in our research, azilsartan activated the Nrf2-mediated intracellular antioxidant system, including HO-1, catalase and SOD1; reduced the level of intracellular ROS, and lead to a decline in osteoclastogenesis.

Several AT1R blockers seemingly activate Nrf2/HO-1 signaling and upregulate the expression of antioxidant enzymes, but further evidence is lacking in BMMs (Fujita et al., 2012; Matsumoto et al., 2014; Saber et al., 2019; Hou et al., 2021). Consistent with previous studies, our results showed that azilsartan activated Nrf2/HO-1 signaling. Further silencing of Nrf2 using siRNAs significantly promoted osteoclastogenesis and upregulated the protein levels of NFATc1 and c-Fos. Surprisingly, azilsartan treatment had little effect on reversing the changes induced by siNrf2, indicating that Nrf2 appears to be a downstream target of azilsartan in our study.

NF-κB and MAPK signaling are critical signaling cascades engaged in osteoclastogenesis. Phosphorylated NF-κB and MAPK signaling proteins activate the transcription factor NFATc1 and promote downstream genes transcription, including *Ctsk*, *Trap*, *and Mmp9* (Boyle et al., 2003; Zhao et al., 2019). We observed that azilsartan treatment inhibited the degradation of IκBα and phosphorylation of P65 in BMMs. Furthermore, MAPK signaling, including phosphorylated P38, phosphorylated ERK, and phosphorylated JNK, was also significantly inhibited by azilsartan treatment in our study. These findings may reveal the molecular mechanism by which azilsartan inhibits osteoclastogenesis; however, the underlying mechanism requires further elucidation.

Recently, the interaction of Nrf2 and NF-κB/MAPK signaling in osteoclasts has been reported (Meng et al., 2021). The Nrf2 activator RTA-408 was reported to inhibit NF-κB signaling in osteoclasts, and STING and Rac-1 may be involved in this process (Sun et al., 2020). Nrf2 deficiency not only increased the activity of the NFATc1 protein and P38 MAP kinase but also increased ROS levels (Hyeon et al., 2013). In our present study, Nrf2 silencing facilitated NF-κB signaling pathway activation by increasing the phosphorylation of P65 and promoting the degradation of IκBα. Simultaneously, Nrf2 silencing reactivated MAPK signaling by facilitating the phosphorylation of P38/ERK/JNK following treatment with azilsartan. Taken together, Nrf2 may function as a potential downstream direct target of azilsartan to inhibit the NF-κB and MAPK signaling pathways.

Osteoporosis is regulated by osteoclasts and osteoblasts (Boyle et al., 2003). The limitation of the present study is that although we have explored the effect of azilsartan on osteoclasts, the effect on osteoblasts was not determined and requires further exploration. Second, we used mice with ovariectomy-induced osteoporosis as the study subjects; however, the cortical bone of mice does not contain the Harvard system and does not adequately reflect Harvard reconstruction. Therefore, we should choose a more suitable animal model in our future studies. Third, Nrf2 is mainly degraded by the ubiquitination process, and further studies are needed to determine whether azilsartan affects the ubiquitination-mediated degradation process.

## Conclusion

**FIGURE 8:**
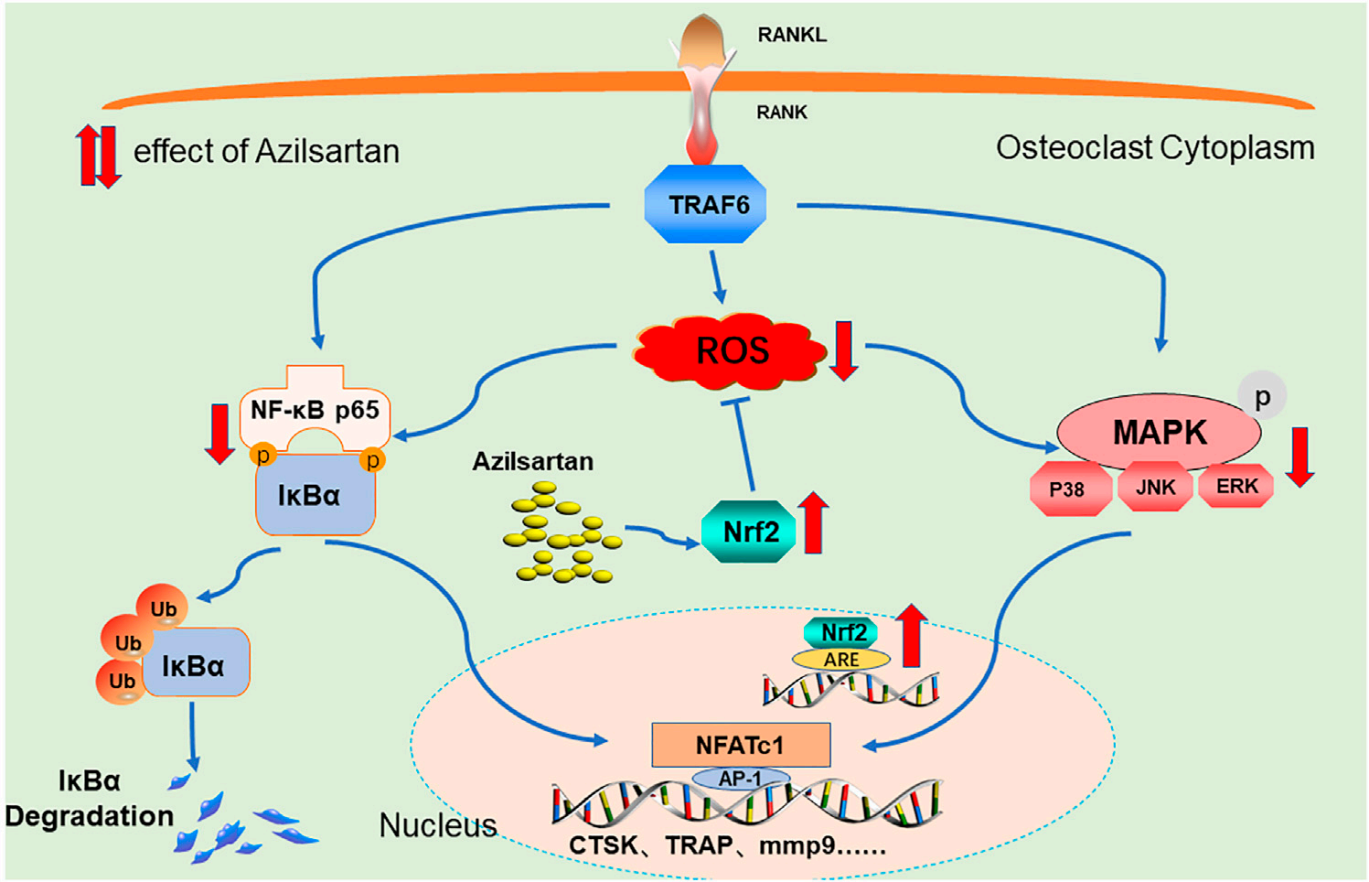
A Proposed scheme of Azilsartan inhibits osteoclastogenesis by suppressing ROS production. Mechanistically, Azilsartan inhibits RANKL-induced activation of NF-κB and MAPK pathways by activating Nrf2 signaling.

In this study, we first found that azilsartan, a novel AT1R blocker, inhibits osteoclastogenesis *in vitro* and attenuates OVX-induced osteoporosis *in vivo* by suppressing ROS production. Mechanistically, azilsartan induced the expression of the antioxidant factor Nrf2, and inhibited the NF-κB/MAPK signaling pathways (Figure 8). Since azilsartan has been approved for clinical use, our findings indicate that azilsartan may be a promising therapeutic agent for the treatment of osteoporosis.

## Data Availability

The original contributions presented in the study are included in the article/Supplementary Material, further inquiries can be directed to the corresponding authors.

## References

[B1] Abdelsaid M. Coucha M. Ergul A. (2014). Cerebrovasculoprotective Effects of Azilsartan Medoxomil in Diabetes. Transl Res. 164 (5), 424–432. 10.1016/j.trsl.2014.06.003 24999268 PMC4250409

[B2] Agidigbi T. S. Kim C. (2019). Reactive Oxygen Species in Osteoclast Differentiation and Possible Pharmaceutical Targets of ROS-Mediated Osteoclast Diseases. Int. J. Mol. Sci. 20 (14), 1. 10.3390/ijms20143576 PMC6678498 31336616

[B3] Asaba Y. Ito M. Fumoto T. Watanabe K. Fukuhara R. Takeshita S. (2009). Activation of Renin-Angiotensin System Induces Osteoporosis Independently of Hypertension. J. Bone Miner Res. 24 (2), 241–250. 10.1359/jbmr.081006 18847324

[B4] Benigni A. Cassis P. Remuzzi G. (2010). Angiotensin II Revisited: New Roles in Inflammation, Immunology and Aging. EMBO Mol. Med. 2 (7), 247–257. 10.1002/emmm.201000080 20597104 PMC3377325

[B5] Benigni A. Corna D. Zoja C. Sonzogni A. Latini R. Salio M. (2009). Disruption of the Ang II Type 1 Receptor Promotes Longevity in Mice. J. Clin. Invest. 119 (3), 524–530. 10.1172/jci36703 19197138 PMC2648681

[B6] Boutros T. Chevet E. Metrakos P. (2008). Mitogen-activated Protein (MAP) Kinase/MAP Kinase Phosphatase Regulation: Roles in Cell Growth, Death, and Cancer. Pharmacol. Rev. 60 (3), 261–310. 10.1124/pr.107.00106 18922965

[B7] Boyle W. J. Simonet W. S. Lacey D. L. (2003). Osteoclast Differentiation and Activation. Nature 423 (6937), 337–342. 10.1038/nature01658 12748652

[B8] Bruzzaniti A. Baron R. (2006). Molecular Regulation of Osteoclast Activity. Rev. Endocr. Metab. Disord. 7 (1-2), 123–139. 10.1007/s11154-006-9009-x 16951988

[B9] Chao Y. Ye P. Zhu L. Kong X. Qu X. Zhang J. (2018). Low Shear Stress Induces Endothelial Reactive Oxygen Species via the AT1R/eNOS/NO Pathway. J. Cell Physiol 233 (2), 1384–1395. 10.1002/jcp.26016 28518223

[B10] Chen K. Qiu P. Yuan Y. Zheng L. He J. Wang C. (2019). Pseurotin A Inhibits Osteoclastogenesis and Prevents Ovariectomized-Induced Bone Loss by Suppressing Reactive Oxygen Species. Theranostics 9 (6), 1634–1650. 10.7150/thno.30206 31037128 PMC6485188

[B11] Chen R. Liu G. Sun X. Cao X. He W. Lin X. (2020). Chitosan Derived Nitrogen-Doped Carbon Dots Suppress Osteoclastic Osteolysis via Downregulating ROS. Nanoscale 12 (30), 16229–16244. 10.1039/d0nr02848g 32706362

[B12] Clézardin P. Coleman R. Puppo M. Ottewell P. Bonnelye E. Paycha F. (2021). Bone Metastasis: Mechanisms, Therapies, and Biomarkers. Physiol. Rev. 101 (3), 797–855. 10.1152/physrev.00012.2019 33356915

[B13] Compston J. E. McClung M. R. Leslie W. D. (2019). Osteoporosis. The Lancet 393 (10169), 364–376. 10.1016/s0140-6736(18)32112-3 30696576

[B14] de Cavanagh E. M. Piotrkowski B. Fraga C. G. (2004). Concerted Action of the Renin-Angiotensin System, Mitochondria, and Antioxidant Defenses in Aging. Mol. Aspects Med. 25 (1-2), 27–36. 10.1016/j.mam.2004.02.006 15051314

[B15] Dong Q. Li Y. Chen J. Wang N. (2021). Azilsartan Suppressed LPS-Induced Inflammation in U937 Macrophages through Suppressing Oxidative Stress and Inhibiting the TLR2/MyD88 Signal Pathway. ACS Omega 6 (1), 113–118. 10.1021/acsomega.0c03655 33458464 PMC7807478

[B16] Fujita H. Fujishima H. Morii T. Sakamoto T. Komatsu K. Hosoba M. (2012). Modulation of Renal Superoxide Dismutase by Telmisartan Therapy in C57BL/6-Ins2(Akita) Diabetic Mice. Hypertens. Res. 35 (2), 213–220. 10.1038/hr.2011.176 22072110 PMC3273720

[B17] Honma M. Ikebuchi Y. Suzuki H. (2021). RANKL as a Key Figure in Bridging between the Bone and Immune System: Its Physiological Functions and Potential as a Pharmacological Target. Pharmacol. Ther. 218, 107682. 10.1016/j.pharmthera.2020.107682 32956720

[B18] Hou N. Li L. R. Shi Y. Y. Yuan W. C. Zhao G. J. Liu X. W. (2021). Azilsartan Ameliorates Ventricular Hypertrophy in Rats Suffering from Pressure Overload-Induced Cardiac Hypertrophy by Activating the Keap1-Nrf2 Signalling Pathway. J. Pharm. Pharmacol. 1, rgab097. 10.1093/jpp/rgab097 34343333

[B19] Hyeon S. Lee H. Yang Y. Jeong W. (2013). Nrf2 Deficiency Induces Oxidative Stress and Promotes RANKL-Induced Osteoclast Differentiation. Free Radic. Biol. Med. 65, 789–799. 10.1016/j.freeradbiomed.2013.08.005 23954472

[B20] Ishii T. Itoh K. Takahashi S. Sato H. Yanagawa T. Katoh Y. (2000). Transcription Factor Nrf2 Coordinately Regulates a Group of Oxidative Stress-Inducible Genes in Macrophages. J. Biol. Chem. 275 (21), 16023–16029. 10.1074/jbc.275.21.16023 10821856

[B21] Kaneko K. Ito M. Fumoto T. Fukuhara R. Ishida J. Fukamizu A. (2011). Physiological Function of the Angiotensin AT1a Receptor in Bone Remodeling. J. Bone Miner Res. 26 (12), 2959–2966. 10.1002/jbmr.501 21887703

[B22] Kanzaki H. Shinohara F. Kajiya M. Kodama T. (2013). The Keap1/Nrf2 Protein axis Plays a Role in Osteoclast Differentiation by Regulating Intracellular Reactive Oxygen Species Signaling. J. Biol. Chem. 288 (32), 23009–23020. 10.1074/jbc.M113.478545 23801334 PMC3743476

[B23] Knopp-Sihota J. A. Cummings G. G. Homik J. Voaklander D. (2013). The Association between Serious Upper Gastrointestinal Bleeding and Incident Bisphosphonate Use: a Population-Based Nested Cohort Study. BMC Geriatr. 13, 36. 10.1186/1471-2318-13-36 23602075 PMC3653746

[B24] Lean J. M. Davies J. T. Fuller K. Jagger C. J. Kirstein B. Partington G. A. (2003). A Crucial Role for Thiol Antioxidants in Estrogen-Deficiency Bone Loss. J. Clin. Invest. 112 (6), 915–923. 10.1172/jci18859 12975476 PMC193670

[B25] Lee N. K. Choi Y. G. Baik J. Y. Han S. Y. Jeong D. W. Bae Y. S. (2005). A Crucial Role for Reactive Oxygen Species in RANKL-Induced Osteoclast Differentiation. Blood 106 (3), 852–859. 10.1182/blood-2004-09-3662 15817678

[B26] Lei J. He M. Xu L. He C. Li J. Wang W. (2021). Azilsartan Prevented AGE-Induced Inflammatory Response and Degradation of Aggrecan in Human Chondrocytes through Inhibition of Sox4. J. Biochem. Mol. Toxicol. 35, e22827. 10.1002/jbt.22827 34051020

[B27] Liu H. Mao P. Wang J. Wang T. Xie C. H. (2016). Azilsartan, an Angiotensin II Type 1 Receptor Blocker, Attenuates Tert-Butyl Hydroperoxide-Induced Endothelial Cell Injury through Inhibition of Mitochondrial Dysfunction and Anti-inflammatory Activity. Neurochem. Int. 94, 48–56. 10.1016/j.neuint.2016.02.005 26879328

[B28] Liu Y. Wang C. Wang G. Sun Y. Deng Z. Chen L. (2019). Loureirin B Suppresses RANKL-Induced Osteoclastogenesis and Ovariectomized Osteoporosis via Attenuating NFATc1 and ROS Activities. Theranostics 9 (16), 4648–4662. 10.7150/thno.35414 31367247 PMC6643439

[B29] Manolagas S. C. (2010). From Estrogen-Centric to Aging and Oxidative Stress: a Revised Perspective of the Pathogenesis of Osteoporosis. Endocr. Rev. 31 (3), 266–300. 10.1210/er.2009-0024 20051526 PMC3365845

[B30] Manolagas S. C. O'Brien C. A. Almeida M. (2013). The Role of Estrogen and Androgen Receptors in Bone Health and Disease. Nat. Rev. Endocrinol. 9 (12), 699–712. 10.1038/nrendo.2013.179 24042328 PMC3971652

[B31] Matsumoto S. Shimabukuro M. Fukuda D. Soeki T. Yamakawa K. Masuzaki H. (2014). Azilsartan, an Angiotensin II Type 1 Receptor Blocker, Restores Endothelial Function by Reducing Vascular Inflammation and by Increasing the Phosphorylation Ratio Ser(1177)/Thr(497) of Endothelial Nitric Oxide Synthase in Diabetic Mice. Cardiovasc. Diabetol. 13, 30. 10.1186/1475-2840-13-30 24485356 PMC3916073

[B32] Meng J. Zhang X. Guo X. Cheng W. Qi X. Huang J. (2021). Briarane-type Diterpenoids Suppress Osteoclastogenisis by Regulation of Nrf2 and MAPK/NF-kB Signaling Pathway. Bioorg. Chem. 112, 104976. 10.1016/j.bioorg.2021.104976 33992967

[B33] Nguyen Dinh Cat A. Montezano A. C. Burger D. Touyz R. M. (2013). Angiotensin II, NADPH Oxidase, and Redox Signaling in the Vasculature. Antioxid. Redox Signal. 19 (10), 1110–1120. 10.1089/ars.2012.4641 22530599 PMC3771549

[B34] Ni S. Qian Z. Yuan Y. Li D. Zhong Z. Ghorbani F. (2020). Schisandrin A Restrains Osteoclastogenesis by Inhibiting Reactive Oxygen Species and Activating Nrf2 Signalling. Cell Prolif 53 (10), e12882. 10.1111/cpr.12882 32871020 PMC7574870

[B35] Nioi P. McMahon M. Itoh K. Yamamoto M. Hayes J. D. (2003). Identification of a Novel Nrf2-Regulated Antioxidant Response Element (ARE) in the Mouse NAD(P)H:quinone Oxidoreductase 1 Gene: Reassessment of the ARE Consensus Sequence. Biochem. J. 374 (Pt 2), 337–348. 10.1042/bj20030754 12816537 PMC1223621

[B36] Novack D. V. (2011). Role of NF-Κb in the Skeleton. Cell Res 21 (1), 169–182. 10.1038/cr.2010.159 21079651 PMC3193402

[B37] Qi D. Liu H. Sun X. Luo D. Zhu M. Tao T. (2020). Pristimerin Suppresses RANKL-Induced Osteoclastogenesis and Ameliorates Ovariectomy-Induced Bone Loss. Front. Pharmacol. 11, 621110. 10.3389/fphar.2020.621110 33628184 PMC7898668

[B38] Raisz L. G. (2005). Pathogenesis of Osteoporosis: Concepts, Conflicts, and Prospects. J. Clin. Invest. 115 (12), 3318–3325. 10.1172/jci27071 16322775 PMC1297264

[B39] Rodan G. A. Martin T. J. (2000). Therapeutic Approaches to Bone Diseases. Science 289 (5484), 1508–1514. 10.1126/science.289.5484.1508 10968781

[B40] Saber S. Khalil R. M. Abdo W. S. Nassif D. El-Ahwany E. (2019). Olmesartan Ameliorates Chemically-Induced Ulcerative Colitis in Rats via Modulating NFκB and Nrf-2/HO-1 Signaling Crosstalk. Toxicol. Appl. Pharmacol. 364, 120–132. 10.1016/j.taap.2018.12.020 30594690

[B41] Sánchez-de-Diego C. Pedrazza L. Pimenta-Lopes C. Martinez-Martinez A. Dahdah N. Valer J. A. (2021). NRF2 Function in Osteocytes Is Required for Bone Homeostasis and Drives Osteocytic Gene Expression. Redox Biol. 40, 101845. 10.1016/j.redox.2020.101845 33373776 PMC7773566

[B42] Sasaki H. Yamamoto H. Tominaga K. Masuda K. Kawai T. Teshima-Kondo S. (2009). Receptor Activator of Nuclear Factor-kappaB Ligand-Induced Mouse Osteoclast Differentiation Is Associated with Switching between NADPH Oxidase Homologues. Free Radic. Biol. Med. 47 (2), 189–199. 10.1016/j.freeradbiomed.2009.04.025 19409483

[B43] Shang Y. (2006). Molecular Mechanisms of Oestrogen and SERMs in Endometrial Carcinogenesis. Nat. Rev. Cancer 6 (5), 360–368. 10.1038/nrc1879 16633364

[B44] Sukumaran V. Tsuchimochi H. Tatsumi E. Shirai M. Pearson J. T. (2017). Azilsartan Ameliorates Diabetic Cardiomyopathy in Young Db/db Mice through the Modulation of ACE-2/ANG 1-7/Mas Receptor cascade. Biochem. Pharmacol. 144, 90–99. 10.1016/j.bcp.2017.07.022 28789938

[B45] Sun X. Xie Z. Hu B. Zhang B. Ma Y. Pan X. (2020). The Nrf2 Activator RTA-408 Attenuates Osteoclastogenesis by Inhibiting STING Dependent NF-Κb Signaling. Redox Biol. 28, 101309. 10.1016/j.redox.2019.101309 31487581 PMC6728880

[B46] Sun Y. X. Xu A. H. Yang Y. Li J. (2015). Role of Nrf2 in Bone Metabolism. J. Biomed. Sci. 22, 101. 10.1186/s12929-015-0212-5 26511009 PMC4625735

[B47] Tan E. M. Li L. Indran I. R. Chew N. Yong E. L. (2017). TRAF6 Mediates Suppression of Osteoclastogenesis and Prevention of Ovariectomy-Induced Bone Loss by a Novel Prenylflavonoid. J. Bone Miner Res. 32 (4), 846–860. 10.1002/jbmr.3031 27813153

[B48] Walsh M. C. Lee J. Choi Y. (2015). Tumor Necrosis Factor Receptor- Associated Factor 6 (TRAF6) Regulation of Development, Function, and Homeostasis of the Immune System. Immunol. Rev. 266 (1), 72–92. 10.1111/imr.12302 26085208 PMC4799835

[B49] Wang S. Ma Q. Xie Z. Shen Y. Zheng B. Jiang C. (2021). An Antioxidant Sesquiterpene Inhibits Osteoclastogenesis via Blocking IPMK/TRAF6 and Counteracts OVX‐ Induced Osteoporosis in Mice. J. Bone Miner Res. 36, 1850–1865. 10.1002/jbmr.4328 33956362

[B50] Xu S. Cao X. Yu Z. He W. Pang Y. Lin W. (2021). Nicorandil Inhibits Osteoclast Formation Base on NF-Κb and P-38 MAPK Signaling Pathways and Relieves Ovariectomy-Induced Bone Loss. Front. Pharmacol. 12, 726361. 10.3389/fphar.2021.726361 34566650 PMC8455841

[B51] Yamashita T. Yao Z. Li F. Zhang Q. Badell I. R. Schwarz E. M. (2007). NF-kappaB P50 and P52 Regulate Receptor Activator of NF-kappaB Ligand (RANKL) and Tumor Necrosis Factor-Induced Osteoclast Precursor Differentiation by Activating C-Fos and NFATc1. J. Biol. Chem. 282 (25), 18245–18253. 10.1074/jbc.M610701200 17485464

[B52] Yang W. Lu X. Zhang T. Han W. Li J. He W. (2021). TAZ Inhibits Osteoclastogenesis by Attenuating TAK1/NF-Κb Signaling. Bone Res. 9 (1), 33. 10.1038/s41413-021-00151-3 34253712 PMC8275679

[B53] Zhang F. Dong Z. Gao S. Chen G. Liu D. (2019). AT1R-Mediated Apoptosis of Bone Marrow Mesenchymal Stem Cells Is Associated with mtROS Production and mtDNA Reduction. Oxid Med. Cell Longev 2019, 4608165. 10.1155/2019/4608165 31772704 PMC6854225

[B54] Zhao X. Ning L. Xie Z. Jie Z. Li X. Wan X. (2019). The Novel P38 Inhibitor, Pamapimod, Inhibits Osteoclastogenesis and Counteracts Estrogen-dependent Bone Loss in Mice. J. Bone Miner Res. 34 (5), 911–922. 10.1002/jbmr.3655 30615802

[B55] Zhao Z. Wang C. Xu Y. Wang X. Jia B. Yu T. (2021). Effects of the Local Bone Renin-Angiotensin System on Titanium-Particle-Induced Periprosthetic Osteolysis. Front. Pharmacol. 12, 684375. 10.3389/fphar.2021.684375 34248634 PMC8264785

[B56] Zheng L. Gao J. Jin K. Chen Z. Yu W. Zhu K. (2019). Macrophage Migration Inhibitory Factor (MIF) Inhibitor 4-IPP Suppresses Osteoclast Formation and Promotes Osteoblast Differentiation through the Inhibition of the NF-Κb Signaling Pathway. Faseb j 33 (6), 7667–7683. 10.1096/fj.201802364RR 30893559

[B57] Zhou L. Zhang S. Bolor-Erdene E. Wang L. Tian D. Mei Y. (2020). NAMPT/SIRT1 Attenuate Ang II-Induced Vascular Remodeling and Vulnerability to Hypertension by Inhibiting the ROS/MAPK Pathway. Oxid Med. Cell Longev 2020, 1974265. 10.1155/2020/1974265 33488923 PMC7791967

