# Supplementary material for: Azilsartan Suppresses Osteoclastogenesis and Ameliorates Ovariectomy-Induced Osteoporosis by Inhibiting Reactive Oxygen Species Production and Activating Nrf2 Signaling
Source: Front Pharmacol. 2021 Nov 26;12:774709. doi: 10.3389/fphar.2021.774709 (PMC8662525; doi:10.3389/fphar.2021.774709)
Supplement: Supplementary file 1 [file DataSheet3.docx]

All pictures and raw data can be obtained by clicking on this link （<https://www.jianguoyun.com/p/DUNT7y0Qz9bqCRiw1YwE>）
