# Supplementary material for: Azilsartan Suppresses Osteoclastogenesis and Ameliorates Ovariectomy-Induced Osteoporosis by Inhibiting Reactive Oxygen Species Production and Activating Nrf2 Signaling
Source: Front Pharmacol. 2021 Nov 26;12:774709. doi: 10.3389/fphar.2021.774709 (PMC8662525; doi:10.3389/fphar.2021.774709)
Supplement: Supplementary file 2 [file DataSheet1.docx]

Supplementary Material

## Supplementary Tables

**Supplementary Table 1: Primer Sequences**

| **Gene (mouse)** | **Forward primer sequence (5’-3’)** | **Reverse primer sequence(5’-3’)** |
| --- | --- | --- |
| *Nfatc-1* | GGAGCGGAGAAACTTTGCG | GTGACACTAGGGGACACATAACT |
| *c-Fos* | GTTCGTGAAACACACCAGGC | GGCCTTGACTCACATGCTCT |
| *Ctsk* | GAAGAAGACTCACCAGAAGCAG | TCCAGGTTATGGGCAGAGATT |
| *Atp6v0d2* | GTGAGACCTTGGAAGACCTGAA | GAGAAATGTGCTCAGGGGCT |
| *Dc-stamp* | GGGGACTTATGTGTTTCCACG | ACAAAGCAACAGACTCCCAAAT |
| *Trap* | CCATTGTTAGCCACATACGG | CACTCAGCACATAGCCCACA |
| *Gapdh* | AGGTCGGTGTGAACGGATTTG | GGGGTCGTTGATGGCAACA |
| *Nrf2* | CTTTAGTCAGCGACAGAAGGAC | AGGCATCTTGTTTGGGAATGTG |
| *Ho-1* | AGGTACACATCCAAGCCGAGA | CATCACCAGCTTAAAGCCTTCT |
| *Catalase* | AAGATTGCCTTCTCCGGGTG | GACATCAGGTCTCTGCGAGG |
| *Sod1* | CACTTCGAGCAGAAGGCAAG | CCCCATACT GATGGACGTGG |
| Si-m-Nfe2l2_009 | | GCATGATGGACTTGGAGTT |

**Supplementary Table 2: Effect of Azilsartan on biochemistry index of mice**

| **Index** | **Sham** | **OVX** | **OVX+Azilsartan** |
| --- | --- | --- | --- |
| ALB (g/L) | 30.35 ±1.11 | 30.6 ±0.82 | 30 ± 4 |
| ALT (U/L) | 38.17 ± 2.36 | 37.5 ± 1.18 | 37.67 ± 1.15 |
| AST (U/L) | 161.7 ± 10.55 | 168 ± 12.19 | 174.7 ± 11.97 |
| ALP (U/L) | 287.2 ± 7 | 277 ± 4 | 284.3 ± 3.5 |
| T-Bil (μmol/L) | 0.3883 ± 0.12 | 0.3117 ± 0.09 | 0.3983 ± 0.11 |

## Supplementary Figures


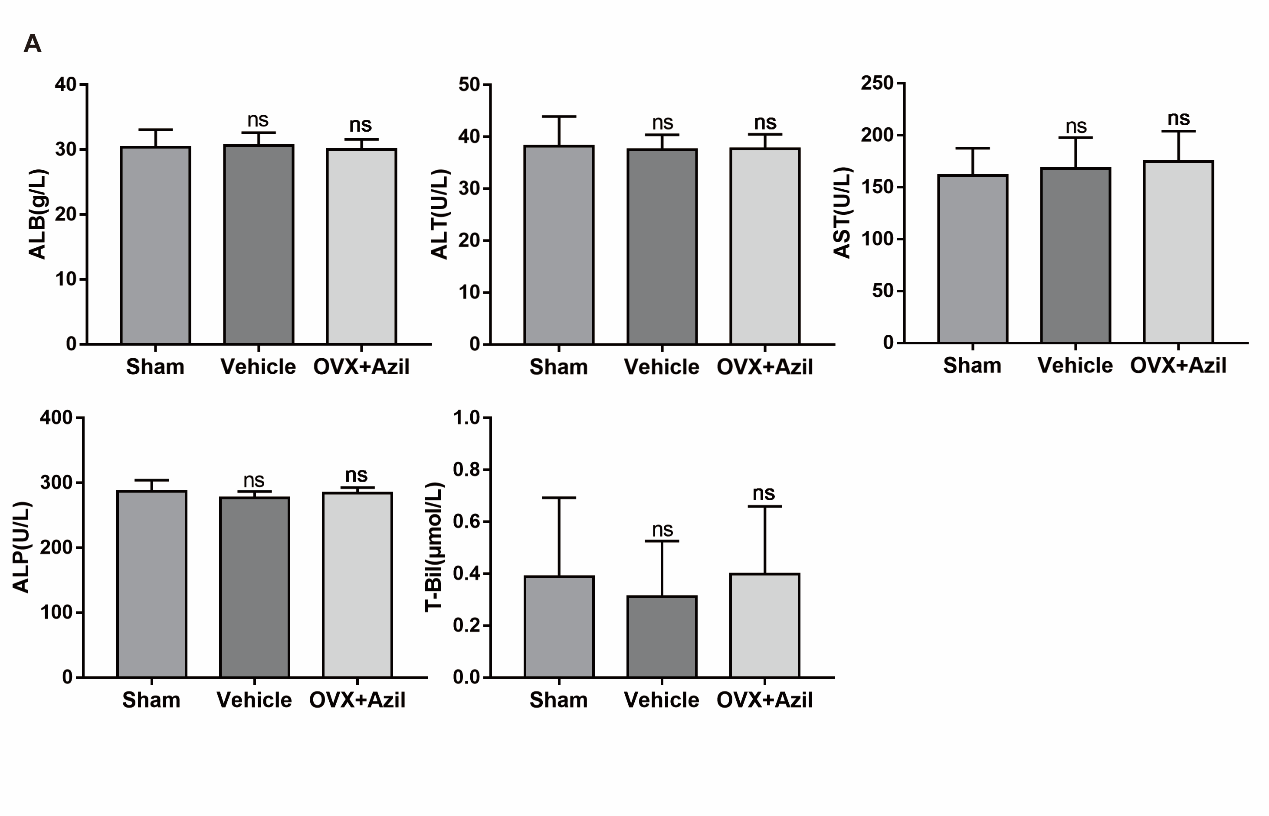


**Supplementary Figure 1. (A)** Azilsartan has no effect on liver functional enzymes in the serum samples of mice (n=6 per group). All data were shown as mean ± SD. (ns, no significance; compared with the sham group)
